# Repeated photoporation with graphene quantum dots enables homogeneous labeling of live cells with extrinsic markers for fluorescence microscopy

**DOI:** 10.1038/s41377-018-0048-3

**Published:** 2018-08-08

**Authors:** Jing Liu, Ranhua Xiong, Toon Brans, Saskia Lippens, Eef Parthoens, Francesca Cella Zanacchi, Raffaella Magrassi, Santosh K. Singh, Sreekumar Kurungot, Sabine Szunerits, Hannelore Bové, Marcel Ameloot, Juan C. Fraire, Eline Teirlinck, Sangram Keshari Samal, Riet De Rycke, Gaëlle Houthaeve, Stefaan C. De Smedt, Rabah Boukherroub, Kevin Braeckmans

**Affiliations:** 1 Laboratory of General Biochemistry and Physical Pharmacy, Faculty of Pharmacy, Ghent University, Ghent, B-9000 Belgium; 2 Centre for Nano- and Biophotonics, Ghent University, Ghent, B-9000 Belgium; 3 VIB-UGent Centre for Inflammation Research, VIB, Ghent, B-9000 Belgium; 4 VIB Bioimaging Core, VIB, Ghent, B-9000 Belgium; 5 Department of Biomedical Molecular Biology, Ghent University, Ghent, B-9000 Belgium; 6 Nanophysics (NAPH), Istituto Italiano di Tecnologia, Genova, 16163 Italy; 7 Biophysics Institute (IBF), National Research Council (CNR), Via De Marini, 6-16149–GE Genova, Italy; 8 Physical and Materials Chemistry Division, CSIR-National Chemical Laboratory, Dr. Homi Bhabha Road, Pune, 411008 India; 9 Academy of Scientific and Innovative Research, Anusandhan Bhawan, 2 RafiMarg, New Delhi, 110 001 India; 10 Univ. Lille, CNRS, Centrale Lille, ISEN, Univ. Valenciennes, UMR 8520-IEMN, Lille, F-59000 France; 11 Biomedical Research Institute, Hasselt University, Agoralaan Building C, Diepenbeek, 3590 Belgium; 12 Centre for Surface Chemistry and Catalysis, KU Leuven, Celestijnenlaan 200F, Leuven, 3001 Belgium; 13 Inflammation Research Center, Image Core Facility, VIB, 9052 Ghent, Belgium; 14 Department of Biomedical Molecular Biology, Ghent University, 9052 Ghent, Belgium; 15 Department of Biomedical Molecular Biology, Ghent University, 9052 Ghent, Belgium; 16 Department of Plant Biotechnology and Bioinformatics, Ghent University, 9052 Gent, Belgium; 17 Univ Lille 1, Univ Lille Nord France, Lab Phys Lasers Atomes & Mol, Villeneuve Dascq, UMR 8523, 59655 France; 18 College of Chemical Engineering, Jiangsu Key Lab of Biomass-based Green Fuels and Chemicals, Nanjing Forestry University (NFU), Nanjing, 210037 China; 19 UMR 8523, Laboratoire de Physique des Lasers, Atomes et Molécules, Université de Lille, Villeneuve d’Ascq, France; 20 IEMN, UMR 8520, Université de Lille, Villeneuve d’Ascq, France

## Abstract

In the replacement of genetic probes, there is increasing interest in labeling living cells with high-quality extrinsic labels, which avoid over-expression artifacts and are available in a wide spectral range. This calls for a broadly applicable technology that can deliver such labels unambiguously to the cytosol of living cells. Here, we demonstrate that nanoparticle-sensitized photoporation can be used to this end as an emerging intracellular delivery technique. We replace the traditionally used gold nanoparticles with graphene nanoparticles as photothermal sensitizers to permeabilize the cell membrane upon laser irradiation. We demonstrate that the enhanced thermal stability of graphene quantum dots allows the formation of multiple vapor nanobubbles upon irradiation with short laser pulses, allowing the delivery of a variety of extrinsic cell labels efficiently and homogeneously into live cells. We demonstrate high-quality time-lapse imaging with confocal, total internal reflection fluorescence (TIRF), and Airyscan super-resolution microscopy. As the entire procedure is readily compatible with fluorescence (super resolution) microscopy, photoporation with graphene quantum dots has the potential to become the long-awaited generic platform for controlled intracellular delivery of fluorescent labels for live-cell imaging.

## Fluorescence microscopy: Labeling cells with laser-based technique

A new laser-based technique that uses graphene nanoparticles for probing subcellular structures and intracellular processes could provide deeper better insights into the role biomolecules and biological pathways play in the metabolic processes of living cells. Although genetic engineering with fluorescent proteins has become the principal method for labeling live cells, the fluorescent proteins only operate across a limited spectral range and are generally not as bright or photostable as traditional external fluorophores. Now, an international team of scientists, led by Kevin Braeckmans and colleagues from Ghent University in Belgium, has developed an innovative laser-based technique that uses graphene nanoparticles, also known as graphene quantum dots, able to deliver a variety of extrinsic cell labels uniformly and efficiently into live cells, opening the door for their use in a wide range of biological and medical applications.

## Introduction

It is imperative to observe subcellular structures as well as intracellular processes to gain insight into the role of biomolecules and biological pathways^1^. While high-quality organic and particulate labels are available for fluorescence (super resolution) microscopy, their use is mainly limited to fixed and permeabilized cells, as they cannot readily permeate through the cell membrane of living cells^2^. This is why genetic engineering with fluorescent proteins has become the predominant labeling method for live cells in the last 15 years. However, apart from the risk of inducing over-expression artifacts, fluorescent proteins come in a limited spectral range and are generally not as bright or photostable as traditional extrinsic fluorophores^3,4^.

In recent years, several intracellular delivery methods have been evaluated for delivering extrinsic labels into live cells for microscopy. Carrier-mediated methods have been proposed in which labels are combined with lipid or polymeric carriers that enter the cells through endocytosis^5,6^. Unfortunately, due to inefficient endosomal escape, the resulting labeling pattern is ambiguous at best, with some of the labels reaching the cytoplasm but the majority remaining trapped inside endosomes^7,8^. An alternative approach is the use of physical or chemical methods that permeabilize the cell membrane, thus bypassing endocytic uptake. For instance, the pore-forming bacterial toxin streptolysin O (SLO) was recently used to deliver exogenous labels in cells^9^. It does, however, require careful optimization of the treatment procedure per cell type, while the pore size is inherently limited to ~100 kDa. Electroporation has also been investigated but is often associated with high cell death and requires transfer of the cells in dedicated recipients for transfection^10,11^. Cell squeezing is a more recent approach based on flowing cells through a microfluidic channel that contains carefully engineered constrictions or obstructions^12^. Shear forces induce pores in the cell membrane, allowing labels to subsequently diffuse into the cells. While this technique is reportedly fast and rather safe for cells, it still requires the cells to be transferred to the microfluidic device and reseeded afterwards for microscopy.

As the need in this area for a broadly applicable intracellular delivery method that is compatible with cell recipients traditionally used for live-cell microscopy remains, we evaluated nanoparticle-assisted photoporation as an emerging new approach for delivering compounds into cells. Plasmonic nanoparticles, usually gold nanoparticles (AuNPs), are incubated with cells so that they make contact with the cell membrane. Laser irradiation is then applied to permeabilize the cell membrane through photothermal effects^13^. One photothermal effect has proven particularly effective for intracellular delivery, which is the generation of vapor nanobubbles (VNBs). Upon irradiation with short (<10 ns) intense laser pulses, plasmonic NPs can become extremely hot such that the surrounding water in the biological tissue evaporates, leading to the formation of VNBs^14,15^. In this case, it is the mechanical force of the expanding and imploding VNBs around each plasmonic nanoparticle that locally punctures the cell membrane, allowing external compounds to diffuse into the cytoplasm. Importantly, being a laser-based technology, it is naturally compatible with light microscopy and can be readily applied to (adherent) cells in culture dishes that are normally used for fluorescence microscopy.

One limitation, however, is that cells incubated with AuNPs can be photoporated only once since AuNPs tend to fragment after applying one laser pulse^16^. Here, we demonstrate for the first time that graphene-based nanoparticles, such as graphene quantum dots (GQDs), are more resistant to pulsed laser irradiation and can form multiple VNBs, thus allowing repeated photoporation of the cells and careful control of the amount of label delivered into the cells. Using these GQDs, we show that fluorescent compounds can be gradually delivered into cells upon application of sequential laser pulses until the desired contrast and label homogeneity are achieved. We demonstrate successful delivery of several types of cell-impermeable fluorescent labels into live HeLa cells, including fluorescently tagged phalloidin, nanobodies, and SNAP-tags. High-quality live-cell imaging is demonstrated with confocal microscopy, TIRF microscopy, and Airyscan super-resolution microscopy. Taken together, these results demonstrate the potential of photoporation sensitized by graphene-based NPs to become a generic platform technology for the labeling of live cells for microcopy.

## Results

### VNB formation threshold and multiple VNB formation with graphene quantum dots

**Fig. 1:**
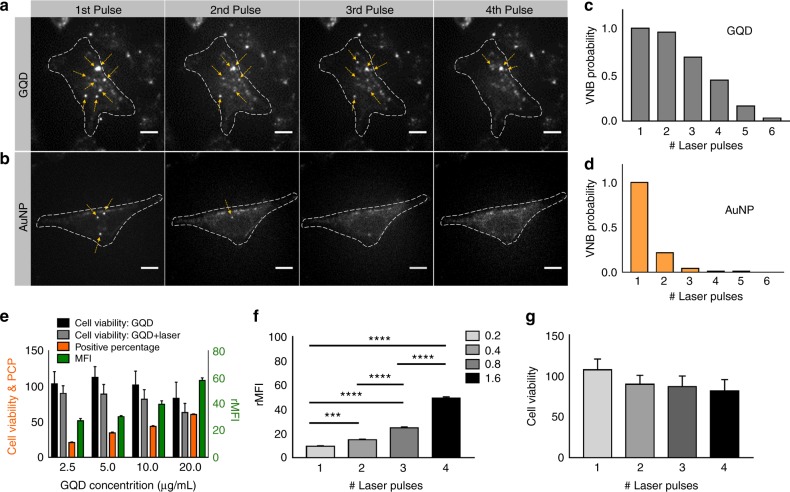
Repeated VNB formation and photoporation of cells with GQDs. HeLa cells were incubated with (**a**) GQDs and **b** AuNPs and irradiated with 4 discrete 7 ns laser pulses at twice the VNB generation threshold. Dark-field images show repeated formation of VNBs (yellow arrows) with each laser pulse for GQDs, but not for AuNPs. The cell boundaries are highlighted by the dashed lines. The scale bar is 20 µm. **c** The VNB formation probability from GQDs upon repeated laser exposure is calculated from the dark-field images (*n* = 25). **d** The same was done for AuNPs, showing that VNBs can only be formed once or, in rare cases, two times per AuNP. **e** Cell viability was measured by the MTT assay before (black bars) and after (gray bars) photoporation with GQDs (2.5 to 20 μg/mL) under the condition of a single-laser pulse. Cells were photoporated with FD10 to quantify the PCP (orange) and the amount of label delivered (green, rMFI relative mean fluorescence intensity). **f** HeLa cells were incubated with 10 µg/mL GQDs and photoporated four times with FD10. The concentration of FD10 was doubled at each step from 0.2 to 1.6 mg/mL to more clearly demonstrate successful subsequent intracellular delivery of FD10. **P < 0.01, ***P < 0.001, ****P < 0.0001. **g** Cell viability was measured after each laser treatment from 1 to 4 laser pulses. Error bars in (**e**, **f**), and **g** stand for three replicates

First, the laser fluence threshold to generate VNBs with 90% certainty was determined to be 0.23 J/cm^2^ for GQDs (Figure S1a) and 0.47 J/cm^2^ for AuNPs (Figure S1b) using 7 ns laser pulses at a wavelength of 561 nm (for experimental details, see the Supplementary Information). All further experiments were performed at approximately twice this threshold value to ensure that VNBs were effectively formed. Repeated application of laser pulses to the GQDs in dispersion confirmed our hypothesis that multiple VNBs can be formed from the same particles, as studied by dark-field microscopy (Suppl. Movie 1). Under dark-field illumination, VNBs scatter light and become visible as brief flashes of light^14,17^. This was not the case for AuNPs (Suppl. Movie 2), which became fragmented after the first laser pulse, as confirmed by transmission electron microscopy images (TEM) (Figure S3) and the UV-vis extinction spectrum (Figure S1c, d). It is indeed known that laser-induced heating can cause AuNPs to melt (1063 ℃ melting point) and/or fragment^18,19^. The exact reason why this phenomenon does not occur for GQDs is not known, but it may be linked to their much higher melting temperature of ~4000–6000 ℃^20^. In comparison, TEM images showed that the size of the GQDs is more gradually reduced upon subsequent laser pulses (Figure S4), most likely due to removal of CO_2_/CO_2_H groups. High-resolution TEM (HRTEM) confirmed the lattice structure of GQDs (Figure S4f). To quantify more precisely how many VNBs can be formed per GQD, we repeated the experiment for nanoparticles incubated with HeLa cells for 30 min, allowing them to bind to the cell membrane. After washing them to remove unbound nanoparticles, cells were irradiated with a series of laser pulses, and the number of visible VNBs after each pulse was quantified among 25 counted cells (Fig. 1a, b, and Suppl. Movies 3 and 4). While AuNPs are able to form VNBs only once or occasionally twice (Fig. 1d), GQDs can do so up to four times (Fig. 1c).

### Photoporation efficiency and cell viability

After the VNB formation threshold was determined, we evaluated how efficient GQDs are in permeabilizing the cell membrane without causing substantial cytotoxicity. After being incubated with different concentrations of GQDs (from 2.5 μg/mL to 20 μg/mL), HeLa cells were photoporated with FITC-dextran 10 kDa (FD10), which was used as a model fluorescent compound to quantify uptake^14^. Flow cytometry analysis showed that the delivery efficiency increases with increasing GQD concentration, both in terms of the positive cell percentage (PCP) and the relative mean fluorescence intensity (rMFI) per cell (Fig. 1e). The same results were also demonstrated by confocal microscopy (Figure S5). A control experiment showed that FD10 delivery does not occur spontaneously in the cells, nor by mere incubation with GQDs (Figure S6). Cell viability was >80% after photoporation for a concentration of GQDs up to 10 μg/mL. Based on these results, it was decided to continue with a GQD concentration of 10 µg/mL for all further experiments. This corresponds to an average of 13 GQDs per cell (Figure S7), as determined from 60 cells using a recently reported microscopy method to visualize carbonaceous nanomaterials by femtosecond (fs) laser irradiation^21^. These images revealed a rather broad size distribution of GQDs on the cells, likely due to aggregation that occurs to some extent after dispersion in the cell medium. This means that VNBs are likely formed not only from individual GQDs but also from agglomerates. To estimate the pore sizes that are formed by GQD photoporation, we next delivered FITC-dextrans of increasing molecular weight into the cells. As shown in Figure S8a and b, 51.2% of cells were labeled with FD10 (hydrodynamic size ~4 nm), while 26.8% of cells were labeled with FITC-dextran of 70 kDa (FD70; hydrodynamic size ~17 nm) and 18.4% of positive cells were labeled with FITC-dextran of 500 kDa (FD500; hydrodynamic size ~31 nm)^22,23^. This shows that pores are created with various sizes up to 30 nm, which is in line with what was previously reported for VNB photoporation with AuNPs^14,24^.

To put photoporation with GQDs into perspective, we performed a comparative experiment in which FD10 was delivered into HeLa cells using traditional photoporation (with AuNPs as sensitizing nanoparticles) and electroporation as a standard transfection method. The results in Figure S9 show that while electroporation yielded >90% positive cells, the rMFI was lower compared to both GQD and AuNP photoporation. The cell viability according to the MTT assay was ~70% after electroporation, which is slightly lower than that for photoporation with GQDs and AuNPs (>80%). Even though electroporation works fairly well, it is important to note that commercial instruments are not compatible with adherent cells grown in recipients for live-cell microscopy. This is the key advantage of photoporation for the application at hand, which is naturally compatible with optical microscopy. Photoporation with AuNPs was more efficient than that with GQDs but lacks the capability of repeated photoporation, as stated before.

The next step was to test if cells could be repeatedly photoporated with FD10 upon repeated pulsed laser irradiation when using GQDs as sensitizing NPs. After each photoporation step, the concentration of FD10 was doubled to establish a stronger concentration gradient and show more clearly successful subsequent intracellular delivery of FD10. As shown in Fig. 1f, the MFI increased significantly with each laser pulse, with only a mild drop in cell viability as measured by the MTT assay (Fig. 1g). Linked to this observation, one can wonder to what extent cytoplasmic content can leak out of the cells following photoporation. To test this, we performed a reverse photoporation experiment. Live cells were first labeled with calcein AM (staining the cytoplasm) followed by treatment with multiple rounds of GQD photoporation (in the absence of any external marker). If leaking occurs, then the calcein AM signal will decrease. Flow cytometry showed a slight fluorescence intensity decrease after each laser treatment (Figure S10), although the decrease after 1 or 2 rounds of photoporation was not statistically significant. Nevertheless, this result shows that a small fraction of the cytoplasm can leak out upon treating cells with photoporation (~25% after 3 × photoporation). We additionally checked to what extent apoptosis is induced by photoporation. Propidium iodide (PI) was used to stain dead cells and DilC_1_(5) to stain live cells. Cells negative for both markers were defined as apoptotic cells^25^. Flow cytometry showed that there was only a small fraction of apoptotic cells, which was not significantly different from the untreated control cells (Figure S11). Together, these results show that GQDs are suitable sensitizing particles for repeated VNB-mediated photoporation of cells.

### Live-cell labeling with extrinsic markers by GQD-mediated photoporation

**Fig. 2:**
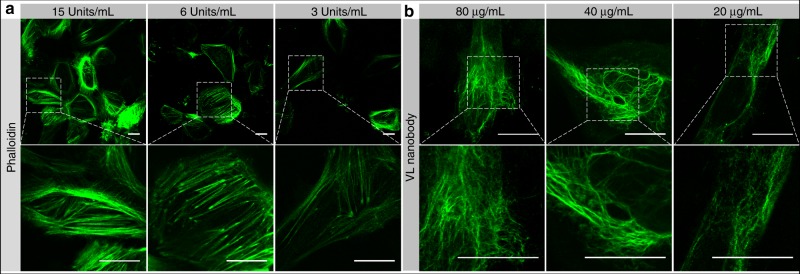
Live HeLa cells were labeled with PL-488 and nanobody by photoporation. **a** Different concentrations (15, 6, and 3 U/mL) of PL-488 were delivered into living HeLa cells by a single photoporation step. Zoomed in images of selected cells are shown in the lower row. **b** Different concentrations (80, 40, and 20 µg/mL) of vimentin-label nanobodies were delivered by photoporation into living HeLa cells. Zoomed in images of selected area are shown in the lower row. Scale bars are 20 µm

Encouraged by the proof-of-concept results, we evaluated if laser-induced photoporation with GQDs is suitable for delivering cell-impermeable exogenous fluorescent labels into live cells. As a first example, we used the phalloidin Alexa Fluor® 488 (PL-488), which can normally stain F-actin in fixed and permeabilized cells only. Figure 2a shows confocal images of live HeLa cells whose actin networks were labeled with different concentration of PL-488 after treating the cells with one laser pulse. Suppl. Movie 5 is a 1 h time-lapse record showing that the cells are alive and that cell activity proceeds. As a novel microscopy fluorescent cell marker, a nanobody was used as the second class of fluorescent labels in this work. We demonstrated successful delivery of a fluorescent vimentin-label nanobody (VL nanobody) targeted to vimentin, an intermediate filament of the cytoskeleton (Fig. 2b). Three different concentrations of VL nanobodies were applied, illustrating that the cells were specifically labeled. A 1 h time-lapse record can be seen in Suppl. Movie 7. To verify the specificity of these stainings, the cells were chemically fixed and permeabilized, followed by co-staining with Alexa Fluor® 647-phalloidin (PL-647) or a primary/secondary anti-vimentin antibody. While the merged images in Figure S12 demonstrate good colocalization, it can be noted that the live-cell staining was not perfectly uniform, an issue that will be resolved in the next section by repeated photoporation.

**Fig. 3:**
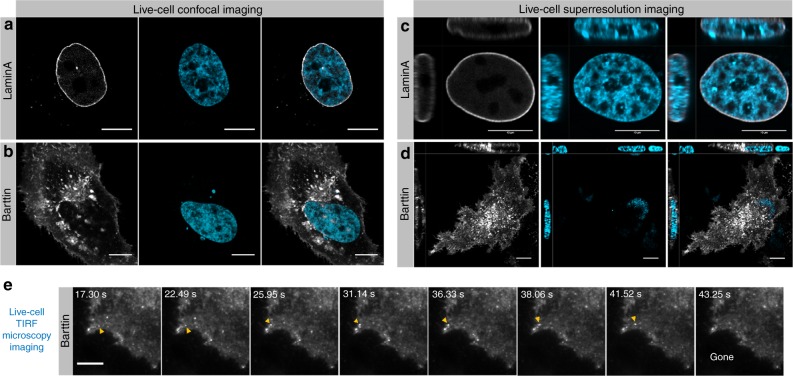
Confocal, Airyscan and TIRF microscopy imaging of live cells after photoporation with SNAP ligand. **a**, **b** Confocal images of live HeLa cells after photoporation with SNAP-Surface® Alexa Fluor® 647 (gray; left). Cells were first transfected with SNAP^LaminA^ or SNAP^Barttin^. The nuclei are stained with Hoechst (cyan; middle). The right column are merged images. **c**, **d** Similarly, transfected and labeled live cells were imaged by Airyscan super-resolution microscopy. The right column are merged images. **e** Time-lapse TIRF microscopy images of Barttin-labeled HeLa cells. The yellow arrowhead indicates vesicle-mediated transport of Barttin. Scale bars are 10 µm

A third class of labels that are increasingly being used, especially for super-resolution microscopy^26–28^, are organic fluorophores linked to a chemical tag that can specifically bind to receptor molecules linked to the protein of interest, such as the SNAP-tag^29^. To test the successful delivery of such ligands, HeLa cells were transfected with DNA encoding for a LaminA or Barttin SNAP-tag fusion protein. LaminA is the inside fibrillar network of the nucleus, while Barttin is a Ka/Kb chloride channel subunit expressed at the cytoplasmic side of the plasma membrane^30,31^. To confirm successful transfection of the fusion proteins, chemically fixed and permeabilized cells were incubated with 1 µM SNAP-surface ligand solution (SNAP-Surface® Alexa Fluor®647). Confocal microscopy imaging of these cells showed a labeled nucleus envelope in the case of LaminA and membrane staining in the case of Barttin, confirming successful transfection (Figure S13). Photoporation was used to deliver the SNAP-tag ligands into the live cells. Confocal images showed the expected staining pattern (Fig. 3a, b), confirming successful live-cell labeling with the SNAP-tag. Additionally, Airyscan microscopy^32^, as an example of super-resolution imaging, was successfully performed, showing the nuclear envelope and Barttin protein (Fig. 3c, d, and Suppl. Movie 8). TIRF images were also recorded of SNAP^Barttin^ live cells, showing vesicle-mediated transport to the inner leaflet of the cell membrane of newly expressed Barttin proteins (Suppl. Movie 9 and Fig. 3e arrowheads).

### Improved cell labeling by repeated photoporation

**Fig. 4:**
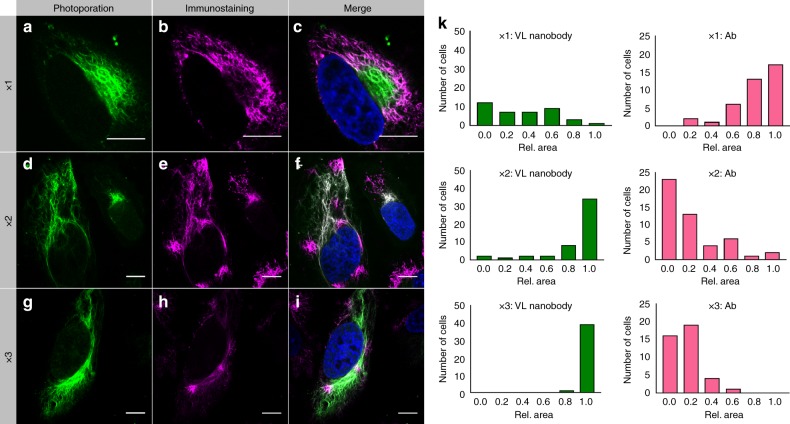
Confocal images of HeLa cells labeled with VL nanobody by repeated photoporation. **a** HeLa cells were photoporated once (1×) with 40 µg/mL VL nanobody Atto 488 (green) and **b** co-stained with anti-vimentin primary Ab and goat anti-mouse secondary Ab (Alexa Fluor® 568) (magenta) after fixation and permeabilization. **c** The merged image of (**a**) and **b**. The same procedure was repeated for cells labeled by **d**–**f** twice repeated (2×) and **g**–**i** third repeated (3×) photoporation steps. Live-cell labeling becomes uniform upon repeated photoporation. The scale bar is 10 µm. **k** Image analysis shows that upon repeated photoporation, the area of green fluorescence increases while the red fluorescence decreases. A total of 39, 49, and 40 cells were analyzed in the 1×, 2×, and 3× repeated photoporation steps, respectively

As noted above for live cells labeled with phalloidin and nanobodies, the intracellular staining was not as uniform compared to fixed cells (see Figure S14). Likely, the probes bind primarily close to the region where the membrane is permeabilized (pores reseal in ~1 min^24,33^), so the more distant areas in the cell receive less label, causing inhomogeneous intracellular labeling. Additional examples are shown in Figure S12 for PL-488 and VL nanobody-labeled live cells after photoporation. We hypothesized that repeated photoporation with GQDs could further enhance the cell labeling homogeneity in live cells. When a second or third VNB is generated, the cell membrane will open again, and more extrinsic markers can enter the cells. Since the first binding places are already occupied, newly entering probes can diffuse further into the cell and label these regions. Consequently, the cells become more uniformly labeled upon repeated photoporation. We demonstrated this principle with the VL nanobody, which caused the most heterogeneous cell labeling. Cells were photoporated once with 40 µg/mL VL nanobodies, fixed, permeabilized, and co-stained with traditional primary/secondary antibody staining against vimentin. A representative confocal image in Fig. 4a–c shows noticeable labeling heterogeneity. The green fluorescence originates from the photoporated VL nanobody, while the magenta fluorescence comes from the labeled secondary antibody that is added after fixation. To further improve the labeling uniformity, we tested two (Fig. 4d–f) and three (Fig. 4g–i) sequential rounds of photoporation. Clearly, the intracellular staining with the VL nanobody (green) became gradually more uniform upon repeated photoporation. In contrast, after fixation of these cells, the labeling with the secondary antibody (magenta) became less uniform as the VL nanobodies already occupied most binding places. We quantified this by measuring the relative green and magenta fluorescence areas in a large number of cells, showing that the green area increases while the magenta area decreases with repeated photoporation (Fig. 4k). After three photoporation steps, the labeling is nearly perfect, with almost all available places in the cell exhibiting green fluorescence.

## Discussion

Here, we have proposed VNB photoporation as a generic technology to enable live-cell labeling with exogenous labels for fluorescence microscopy. Instead of the traditionally used AuNPs, we have shown that repeated VNB photoporation is possible with GQDs as sensitizing particles. The formation of multiple VNBs from the same set of GQD particles was shown directly with dark-field imaging, as well as by flow cytometry showing increasing uptake of FITC-dextran upon repeated photoporation. TEM images also demonstrate that GQDs are more resistant to the pulsed laser irradiation than AuNPs, which fragment after the first pulse. To demonstrate the broad applicability of GQD-sensitized VNB photoporation, we successfully delivered three different classes of cell-impermeable exogenous labels into live cells. Phalloidin is an example of a small organic molecule that can be used to label specific subcellular structures, the actin skeleton in this case. Nanobodies are biomolecules that have received increasing attention as a smaller replacement for antibodies for immunostaining cells^9,34^. The SNAP-tag was finally included as an example of an affinity tag that is widely used for high-resolution microscopy imaging. Together, the results show that VNB photoporation is suited to delivering virtually any exogenous label, and in future research, it may be of interest to attempt to utilize even nanoparticle-type labels, such as quantum dots^35^ and upconversion nanoparticles^36^. After successful labeling, heterogeneity was noted within cells, as labels enter through a number of discrete pores in the cell membrane. However, by photoporating the cells multiple times, we could show that the labeling homogeneity was markedly improved. Being compatible with light microscopy and normally used cell recipients, this technique should find rapid acceptance in the field and offers a solution to the long-standing problem of using high-quality extrinsic labels for live-cell microscopy.

Laser-induced photoporation provides a physical and therefore universal approach to overcome the cell membrane barrier for delivering exogenous cell labels directly into cytoplasm. Contrary to microinjection^37^ and the related “nanoblade” technique^38^, nanoparticle-sensitized photoporation can treat cells under high throughput. Even though we used a laser with a rather low pulse repetition rate (20 Hz), treating a well of a 96-well plate containing ~30,000 cells did not take more than 3–4 min. Importantly, the photoporation protocol is completely compatible with optical microscopy and the types of cell substrates typically used for it. This is a marked advantage over other recently proposed technologies for cell labeling, such as cell squeezing^12^, which only works for cells in suspension, or the biophotonic laser-assisted surgery tool (BLAST)^39^ that requires cells to be seeded on a special micro-machined substrate. While the latter does allow large cargo, such as entire chromosomes, to more easily enter cells, this is not really needed for the relatively small probes for microscopy. The present study was demonstrated on HeLa cells as a proof-of-concept. Although HeLa cells are rather robust cells, this live-cell delivery technique can be readily applied to a broad variety of cell types, including more sensitive ones. For instance, in a previous work of ours, we already demonstrated the application of photoporation (with AuNPs) on primary hard-to-transfect T cells^40^ and densely cultured hippocampal neuron cells isolated from mice^41^.

GQDs were used for photoporation in this work, which are a graphene-based nanomaterial that has attracted considerable interest for a wide variety of bio-applications, such as biosensing, drug delivery, and bioimaging^42,43^. The usage of GQDs as sensitizing nanoparticles, allowing repeated VNB photoporation, turned out to be crucial to achieving uniform labeling of live cells. Here, we report for the first time that these graphene-based nanomaterials can form multiple VNBs upon repeated pulsed laser irradiation, which is a distinct advantage over the traditionally used AuNPs for photoporation. While still only 3–4 VNBs can be formed per particle, this proved to be sufficient for the application. It is to be noted that, in one report, it was found that the fragmentation of AuNPs could be avoided by irradiation with fs laser pulses at off-resonant wavelengths^16^. While an interesting observation, such fs lasers are much more expensive than ns pulsed lasers, which are available at a fraction of the cost and, therefore, much more likely to be of practical relevance. The GQDs used here are just one example of the many types of graphene-based nanomaterials currently available. Indeed, we have also been able to generate multiple VNBs with reduced graphene oxide nanosheets (data not shown). This shows that graphene-type materials offer generic value for photoporation and live-cell labeling in particular. Future research should focus on exploring the pros and cons of the various types available. For the GQDs used here, it will be of interest to provide further modifications, such as functionalization with polyethylene glycol in order to enhance the colloidal stability and prevent them from forming aggregates in cell medium, which can cause substantial toxicity^44^. It may be worthwhile to try to stabilize GQDs in future work to further improve their performance and safety.

## Materials and methods

### Cell culture and transfection

HeLa cells (ATCC® CCL-2™) were cultured in DMEM/F-12 (Gibco-Invitrogen) supplemented with 10% heat-inactivated fetal bovine serum (FBS, Biowest), 2 mM glutamine (Gibco-Invitrogen), and 100 U/mL penicillin/streptomycin (Gibco-Invitrogen). Cells were passaged using DPBS (Gibco-Invitrogen) and trypsin-EDTA (0.25%, Gibco-Invitrogen). HeLa cells were cultivated in a humidified tissue culture incubator at 37 °C and 5% CO_2_. All cell culture products were purchased from Life Technology unless specifically stated otherwise. Transient transfection was performed with Lipofectamine 2000 (Life Technologies), following the manufacturer’s instructions. After one day of incubation, the cells were washed three times with PBS and were ready for photoporation.

### VNB generation and detection

A homemade setup including an optical and electronic timing system was used to generate and detect the VNBs, as reported previously^14^. In brief, a 7-ns pulsed laser tuned to a wavelength of 561 nm (Opolette HE 355 LD, OPOTEK Inc.) was applied to excite the AuNPs and GQDs in order to generate VNBs. This wavelength was chosen as it is compatible with the fluorescence filters that are in place in our setup for fluorescence microscopy and because it allows for a direct comparison with photoporation using 70 nm AuNPs as sensitizing particles, which have a plasmon peak at ~560 nm. The VNBs can be visualized using dark-field microscopy. An electronic pulse generator (BNC575, Berkeley Nucleonics Corporation) was used to generate single-laser pulses and trigger the camera (EMCCD camera, Cascade II: 512, Photometrics) to record images before and during VNB generation. The laser pulse energy was measured by an energy meter (J-25MB-HE&LE, Energy Max-USB/RS sensors, Coherent). The laser fluence was calculated as the pulse energy of a single-laser pulse divided by the laser beam area (150 µm diameter). Suspensions of 5 µg/mL GQDs and 0.2 µg/mL AuNPs in serum reduced Opti-MEM were prepared to detect VNBs in solution. To detect VNBs on cells, 60,000 HeLa cells were seeded in a 50 mm glass-bottom dish (MatTek Corporation) one day in advance. The cells were incubated for 30 min with 10 µg/mL GQDs or 0.4 µg/mL AuNPs dispersed in Opti-MEM. Unbound particles were washed with DPBS, after which fresh cell culture medium was added. After the experiment, the VNBs were counted in each image and the results reported in a graph that expresses the number of VNBs as a function of the laser fluence.

### Intracellular delivery of FD10 by photoporation

To prove the successful intracellular delivery and determine the appropriate GQD concentration, FD10 was used as a model fluorescent label for photoporation. A 96-well plate was seeded with 15,000 HeLa cells per well one day in advance. Before laser treatment, cells were incubated with a GQD suspended in Opti-MEM solution for 30 min. FD10 was dispersed in cell culture medium (CCM) at 1 mg/mL as a working solution. The CCM was discarded, and 100 µL of FD10 working solution was added into the wells before laser treatment. As the photoporation laser beam has a diameter of 150 µm, a scanning procedure was used to treat all cells within each well. The sample was scanned through the photoporation laser beam (20 Hz pulse frequency) using an electronic microscope stage (HLD117, Prior Scientific, USA). The scanning speed was 2.1 mm/s, and the distance between subsequent lines was 0.1 mm to ensure that each cell received a single-laser pulse. The photoporation of one well took ~4 min. Afterwards, the fluorophore solution was removed, and the cells were washed gently by DPBS and supplemented with fresh cell medium. The amount of intracellular FD alongside the cytotoxicity was measured as explained below. For multiple photoporation rounds, the fluorophore solution was renewed, and the procedure was repeated as described above.

### Confocal microscopy imaging

Microscopy imaging was performed on a laser scanning confocal microscope (C1 si, Nikon) using a ×10 objective (CFI Plan Apochromat, Nikon) and a ×60 oil immersion objective (CFI Plan Apo VC, Nikon). The following lasers were used for excitation: a 405 nm continuous wave laser (Melles Griot 56ICS/S2695) for DAPI and Hoechst; a 488 nm continuous wave laser (Coherent Sapphire) for FD10, ATTO 488 and Alexa Fluor® 488; a 561 nm continuous wave laser (Melles Griot 85-YCA-010) for Alexa Fluor® 568 and a 640 nm continuous wave laser (Melles Griot 56ICS/S2695) for Alexa Fluor® 647. After photoporation, cells were incubated with 1 µg/mL Hoechst (Life Technology, Belgium) in CCM for 15 min at 37 °C. After the cells were washed twice with DPBS, time-lapse recordings were performed on a spinning disk confocal microscope (Nikon eclipse Ti-e inverted microscope, Nikon) equipped with an MLC 400 B laser box (Agilent technologies), a Yokogawa CSU-22 Spinning Disk scanner (Andor) and an iXon ultra EMCCD camera (Andor Technology, Belfast, UK). HeLa cells were imaged in a stage-top cell incubator (37°C with 5% CO_2_ supplied, Tokai Hit) for 1 h with a time interval of 3 min using a ×60 oil immersion objective lens (CFI Plan Apo VC 60 × oil, Nikon, Japan).

### Live-cell labeling with phalloidin and vimentin-label nanobody (VL nanobody)

HeLa cells were seeded in a glass-bottom 96-well plate (Greiner Bio-One) at 15,000 per well one day in advance. Cells were washed once with 200 µL of DPBS, and the same volume of Opti-MEM GQD solution was added into the wells. Cells were incubated with the GQDs for 30 min in a 37 °C incubator. After incubation, cells were washed again with DPBS and supplied with fresh CCM. The working solutions as described in the results section were made from the stock solution of 40 U/mL phalloidin (PL-488, Invitrogen) and 0.4 mg/mL VL nanobody (ChromoTek) in DPBS. Before laser treatment, the cell medium was replaced by 50 µL of PL-488 or VL nanobody working solution. After the photoporation procedure, three washing steps were performed, and the cells were replenished with fresh CCM for confocal imaging. For multiple photoporation steps, the cells were again incubated with the PL-488 or VL nanobody stock solution, and the same process was repeated.

### Live-cell labeling with the SNAP-tag

After transfection with SNAP-tag pDNA, the cell-impermeable SNAP-tag ligand (SNAP-Surface® Alexa Fluor® 647, NEW ENGLAND Biolabs) was delivered into the cells by photoporation. The SNAP ligand was dissolved in CCM at 2 µM and added to the cells prior to starting the photoporation procedure. After photoporation, the nuclei were stained by incubating the cells with 1 µg/mL Hoechst (Life Technology) in CCM for 15 min at 37 °C. After removing the Hoechst solution, cells were washed once, and the wells were refilled with CCM. Airyscan super resolution and TIRF imaging were performed as explained below.

### Airyscan super-resolution imaging

Images were collected on an LSM880 Airyscan (Carl Zeiss) system with a 63 × DIC M27 objective (PlanApo NA: 1.4, oil immersion, Carl Zeiss) using the operating software ZEN blue 2.3. DAPI and Alexa Fluor® 647 were excited with a 405 nm diode laser and a HeNe 633 nm laser, respectively. The filter sets BP 555-620 + LP645 for Alexa Fluor® 647 and BP 420-480 + BP 495-550 for DAPI were used in conjunction with the Airyscan detector in the super-resolution mode. Thirty z-slices were taken with a z-interval of 0.25 µm. A 3D deconvolution step followed by a Wiener filter was carried out post-acquisition. Orthogonal slices of the z-stack were made in Volocity 6.3.0 (Perking Elmer). A time-lapse movie was made on one focal plane with an imaging frequency of 1 min for a total duration of 30 minutes. In this case, a 2D deconvolution step followed by a Wiener filter was carried out post-acquisition.

### TIRF microscopy imaging

A total of 150,000 HeLa cells were seeded in a high glass-bottom 35-mm µ-Dish (Ibidi) 24 h before imaging. Images were collected on a Zeiss Observer 1.1 microscope (Carl Zeiss) with a ×100 TIRF oil immersion objective (PlanApo, NA: 1.46, Carl Zeiss). Alexa Fluor® 647 was visualized with a 639 nm diode laser at an incident angle of −70°, which allows selective excitation of molecules within ~95 nm of the cover glass. The filter set 77 HE GFP/ mRFP/ Alexa 633 (Carl Zeiss) was used in conjunction with an EMCCD Image MX2 camera (Hamamatsu). Image acquisition was performed at an exposure time of 1 s and an EM gain of 50. Time-lapse imaging was carried out with a frequency of 1 s for a total time of 90 s.

## Electronic supplementary material

Supplementary Information

Suppl. Movie 1. Dark-field imaging of VNBs from discrete AuNPs in Opti-MEM

Suppl. Movie 2. Dark-field imaging of VNBs from discrete GQDs in Opti-MEM

Suppl. Movie 3. Dark-field confocal microscopy imaging of VNBs from AuNPs imbedded in a living HeLa cell membrane

Suppl. Movie 4. Dark-field confocal microscopy imaging of VNBs from GQDs imbedded in a living HeLa cell membrane

Suppl. Movie 5. Time-lapse confocal microscopy imaging of living cells labeled of actin filament with PL-488 (green) delivered by photoporation

Suppl. Movie 6. Z-stack imaging of a living HeLa cell labeled with VB nanobody after 1× photoporation

Suppl. Movie 7. Time-lapse confocal imaging of live cells labeled with VB nanobody delivered by photoporation

Suppl. Movie 8. Time-lapse Airyscan superresolution microscopy imaging of living cells

Suppl. Movie 9. Time-lapse TIRF microscopy imaging of living cells
