# Supplementary material for: Repeated photoporation with graphene quantum dots enables homogeneous labeling of live cells with extrinsic markers for fluorescence microscopy
Source: Light Sci Appl. 2018 Aug 8;7:47. doi: 10.1038/s41377-018-0048-3 (PMC6106998; doi:10.1038/s41377-018-0048-3)
Supplement: Supplementary file 1 — Supplementary Information [file 41377_2018_48_MOESM1_ESM.docx]

Supplementary Information (ESI)

**Repeated photoporation with graphene quantum dots enables homogeneous labeling of live cells with extrinsic markers for fluorescence microscopy**

Jing Liu^1,2^, Ranhua Xiong^1,2^, Toon Brans^1,2^, Saskia Lippens5^3,4,5^, [Eef Parthoens](http://www.vib.be/whoiswho/Pages/Eef-Parthoens.aspx?lang=en)^3,4,5^, Francesca Cella Zanacchi^6^, Raffaella Magrassi^6,7^, Santosh K. Singh^8,9^, Sreekumar Kurungot^8,9^, Sabine Szunerits^10^, Hannelore Bové^11,12^, Marcel Ameloot^11^, Juan C. Fraire^1,2^, Eline Teirlinck^1,2^, Sangram Keshari Samal^1,2^, Riet De Rycke^13,14^, Gaëlle Houthaeve^1,2,15^, Stefaan C. De Smedt^1,16^, Rabah Boukherroub^10^, Kevin Braeckmans^1,2,17^

1 Laboratory of General Biochemistry and Physical Pharmacy, Faculty of Pharmacy, Ghent University, Ghent, B-9000, Belgium

2 Centre for Nano- and Biophotonics, Ghent University, Ghent, B-9000, Belgium

3 VIB-UGent Centre for Inflammation Research, VIB, Ghent, B-9000, Belgium

4 VIB Bioimaging Core, VIB, Ghent, B-9000, Belgium

5 Department of Biomedical Molecular Biology, Ghent University, Ghent, B-9000, Belgium

6 Nanophysics (NAPH), Istituto Italiano di Tecnologia, Genova 16163, Italy

7 Biophysics Institute (IBF), National Research Council (CNR), Via De Marini, 6 - 16149 – GE, Italy

8 Physical and Materials Chemistry Division, CSIR-National Chemical Laboratory, Dr. Homi Bhabha Road, Pune 411008, India

9 Academy of Scientific and Innovative Research, Anusandhan Bhawan, 2 RafiMarg, New Delhi 110 001, India

10 Univ. Lille, CNRS, Centrale Lille, ISEN, Univ. Valenciennes, UMR 8520 - IEMN, Lille, F-59000, France

11 Biomedical Research Institute, Hasselt University, Agoralaan Building C, Diepenbeek 3590, Belgium

12 Centre for Surface Chemistry and Catalysis, KU Leuven, Celestijnenlaan 200F, Leuven 3001, Belgium

13 Center for Inflammation Research, VIB, Ghent, Belgium and Department of Biomedical Molecular Biology, Ghent University, 9052 Ghent, Belgium

14 Center for Plant Systems Biology, VIB, Ghent, Belgium and Department of Plant Biotechnology and Bioinformatics, Ghent University,  9052 Gent, Belgium.

15 Laboratory of Cell Biology and Histology, Department of Veterinary Sciences, University of Antwerp, Antwerp, Belgium

16 College of Chemical Engineering, Jiangsu Key Lab of Biomass-based Green Fuels and Chemicals, Nanjing Forestry University (NFU), Nanjing 210037, PR China

17 Université de Lille, IEMN UMR 8520 and Lab. Phys. Lasers Atomes & Mol. UMR 8523, Villeneuve d’Ascq, France

Corresponding author: K. Braeckmans, Telephone: 0032 92648098, Fax: 0032 92648189, Email: Kevin.Braeckmans@UGent.be

**Plasmid construction**

The pSNAPf-C1 plasmid of the LaminA SNAP-tag was purchased from Addgene (LaminA-C-18, #58193 addgene). The Barttin SNAP-tag plasmid was constructed as follows. Standard methods were used to insert the human Barttin coding sequence into the pSNAPf vector (NEB) N-terminal of the SNAPf sequence. The Barttin sequence was amplified from a pCDNA3.1 construct using primers designed to encode the 5′ and 3′ termini of Barttin and containing the recognition sequences of NheI and EcoRI restriction endonucleases, respectively. The pSNAPf vector and the PCR product were cut with NheI and EcoRI (ThermoFisher Scientific) and the Barttin sequence was inserted by ligation. The construct was verified by Sanger sequencing.

**VNB formation threshold determination**

40 nm GQDs and 70 nm AuNPs were dispersed in serum-reduced Opti-MEM to determin the laser fluence threshold for VNB formation. The particles were irradiated by 7 ns laser pulses with a wavelength of 561 nm, which is suitable to excite both particles according to their UV-Vis extinction spectrum (Fig. S1a and S1b). The laser fluence threshold to generate VNB can be determined with dark-field microscopy by counting the number of visible VNBs within the laser spot (150 µm laser beam diameter) for increasing laser pulse energies.^1,2^ Several dark field images are shown in Fig. S2, indicating that more VNBs are formed for increasing laser pulse fluences. The VNB threshold, defined as the laser fluence at which VNBs are formed with 90% certainty^3^, was ~230 mJ cm^-^² for GQDs (Fig. S1c) and ~470 mJ cm^-^² for AuNPs (Fig. S1d). All further experiments will be performed at approximately twice the VNB threshold to be certain that VNBs are effectively formed.

**Ultraviolet-visible extinction spectrophotometry (UV-vis) and transmission electron microscopy (TEM)**

UV-vis extinction spectra and TEM images of GQDs and AuNPs were acquired before and after laser irradiation. UV-vis extinction spectra were measured by NanoDrop 2000c Spectrophotometer (ThermoFisher Scientific) with 20 µg/mL of GQDs and 50 µg/ml of AuNPs dissolved in DI water. AuNPs and GQDs dispersions were irradiated in 96-well plates by pulse laser, as explained below. For TEM imaging a 50-µL drop of the respective sample was blotted on formvar/ C-coated hexagonal copper grids (EMS G200H-Cu) for 20 min and washed 5 times in double distilled H_2_O. TEM images were recorded with a JEM 1400plus transmission electron microscope (JEOL, Tokyo, Japan) operating at 60 kV.

**GQDS synthesis method**

The synthesis of GQDs was achieved according to a procedure reported recently. In Brief, reduced graphene oxide (rGO) was synthesized by the treatment of graphene oxide (GO) with hydrazine monohydrate. GO was synthesized by following a modified Hummer’s method as indicated in reference.^4^ To synthesize small pieces of GQDS, 100 mg of rGO powder was dispersed in 100 mL of 30% H_2_O_2_ and ultrasonicated for 30 min. The obtained uniformly dispersed solution was kept refluxing for 12 h at 60°C. The resulting solution was filtered and GQDs separated from porous reduced graphene. The obtained GQDSs were further dialysed to remove excess H_2_O_2_ and to separate rGO from small sized GQDs. The purified GQDs were dissolved in DI water to a final concentration of 1 mg/mL.

**Detection of cell-associated GQDS**

For photoporation, it is essential that the sensitizing nanoparticles are associated with the cell membrane. The number of GQDs per cell was determined by a recently reported microscopy method capable of detecting carbon-based nanomaterials by fs laser irradiation.^5^ HeLa cells were seeded for 15000 cells per well in a glass bottom 96-well plate (Greiner Bio-One). One day later, cells were washed once with DPBS, incubated with 10 µg/mL opti-MEM GQDs solution for 30 min at 37°C and washed three more times with DPBS to remove unbound GQDs. For visualization of the cell membrane, the cells were subsequently incubated for 30 min with 12.5 µM CellTracker^TM^ Green CMFDA (Life Technologies) and again washed one more time with DPBS afterwards. The cells were fixed with 4% paraformaldehyde and washed three times with DPBS. Before confocal imaging, all wells were aspirated and mounted using 150 µL Immu-Mount^TM^ (Thermo Fisher Scientific).

Images were acquired using a Zeiss LSM510 META NLO scan head mounted on an inverted laser-scanning microscope (Zeiss Axiovert 200M, Zeiss) and a 40×/1.1 water immersion objective. CellTracker^TM^ Green was excited by using the 488-nm air-cooled Argon ion laser (LASOS Lasertechnik GmbH). The emission was filtered by using a band-pass filter of 500 – 550 nm. GQDs were visualized by femtosecond pulsed laser excitation (~ 4 mW average laser power at the sample, 810 nm, 150 fs, 80 MHz, MaiTai DeepSee, Spectra Physics, USA) and filtering of the emission signal by a 425 – 480 nm band-pass filter in the non-descanned mode. Three-dimensional z-stacks were acquired throughout the cells using the AIM 4.2 software (Zeiss). The images were processed with the image-processing program Fiji/ImageJ (ImageJ v1.47, open source software, <http://fiji.sc/Fiji>) to quantify the number of GQDs per cell.

**Flow cytometry and MTT assay**

Following photoporation with FD, cells were detached by trypsin-EDTA treatment and collected by centrifugation. After the cells were re-suspended in flow buffer (PBS supplemented with 5% FBS), the samples were measured by flow cytometry (FACS Calibur, BD). 8000 cells were set to be measured for one sample. A 488-nm laser was used to excite FD10 and the fluorescence intensity was recorded in the 530/30 channel. Additionally cytotoxicity was measured by the MTT assay. Cells after photoporation were put back in the incubator to recover for 2 h. Afterwards, 30 μL of 5 mg/mL 3-(4,5-dimethylthiazol-2-yl)-2,5-diphenyltetrazolium bromide (MTT, Sigma) solution was added to each well. After incubation for 3 h at 37°C, the solution was discarded and 100 μL dimethyl sulfoxide (Sigma) was added into each well to lyse the cells. The formazan crystals in cells were completely dissolved in the solution after shaking for 30 min. The absorbance of the solution in each well was measured at 570 nm and 650 nm using an Envision Xcite multilabel reader (PerkinElmer LAS). Positive and negative controls were also measured to calculate the final cell viability. All data is expressed as the mean ± SD (n = 3).

**Apoptosis measurement**

After multiple photoporation experiments, the cells were trypsinized and re-suspend in flow buffer. 50 nM DilC_1_(5) iodide (Santa Cruz Biotechnology) and 1 µg/mL propidium iodide (PI, Sigma-Aldrich) were added into the solution. The cells were incubated in the flow tube at 37 ℃ for 30 min. Afterwards, the cells were measured by flow cytometer.

**Electroporation**

HeLa cells were trypsinized and counted as described before. 200000 cells were suspended in 200 µL cell culture medium (CCM) and transferred into the electroporation cuvette. 100 µL of 3 mg/mL FD10 was added before electroporation. The electroporator (BIO-RAD, Gene Pulser^®^ II) was set according to the manufacturer’s instruction for HeLa cells (250 V voltage, adb 200 µF capability). After the electric pulse, cells were transferred into an 1.5 mL Eppendorf tube and centrifuged at 11000 rpm for 5 min. Then cells were re-suspended in CCM and centrifuged. This washing step was repeated for three times. The cells were seeded at 15000 cells per well in a 96-well plate and the MTT assay was performed after 4 h incubation. Flow cytometry was performed after the washing step. Control cells were incubated with the same amount of FD10 for the same period as electroporated cells.

**Photoporation induced cytoplasmic leakage**

HeLa cells were seeded in 96-well plates as described before. Calcein AM (Life Technology, Belgium) was diluted in CCM for 10 ng/mL and incubated with the cells at 37 ℃ for 15 min. The cells were then washed once with PBS and treated with (repeated) photoporation without adding any external fluorophore. The fluorescent intensity of the cells after photoporation was measured by flow cytometer.

**Immunostaining in fixed cells**

Cells were rinsed with DPBS and fixated with 4% paraformaldehyde (Sigma-Aldrich) for 15 min at room temperature. After fixation, cells were permeabilized by a PBT solution which consists of 0.02% Triton X-100 and 0.5% BSA dissolved in 1× DPBS and adjusted to a pH of 7.2-7.4. Cells were washed with PBT for 4 times during 5 min each and blocked with 5% goat serum (Invitrogen, Belgium) for 1h at room temperature. PL-647 (Invitrogen) and monoclonal mouse anti-vimentin Ab (Dako) were dissolved in the PBT solution in a final concentration of 3 U/mL and 0.52 µg/ml respectively and added to the cells for 16 hours at 4°C. Cells stained with PL-647 were washed 4 times for 5 min with the PBT solution. Cells incubated with the primary anti-vimentin Ab received an additional incubation step of 30 min. Next, incubation with 10 µg/mL of the fluorescently labeled goat anti-mouse secondary antibody (Alexa Fluor® 568, Abcam) diluted in PBT was performed for 2 hours at room temperature. Next cells were washed 4 times for 5 min with PBT. Cells were mounted with vectashield containing DAPI (VECTOR LABORATORIES) to counterstain the nucleus.

**SNAP-tag labeling in fixed cells**

After transfection with SNAP-tag pDNA, cells were fixated by 4% paraformaldehyde at room temperature for 15 min. The same permeabilization step was performed on the cells after fixation. DPBS was used instead of the blocking buffer after discarding the PBT solution to wash the cells twice, followed by incubation with 1 µM SNAP ligand dissolved in DPBS. Afterwards the cells were put at 4°C overnight. On the second day, cells were washed for four times with PBT for 5 min. Vectashield with DAPI was added to the cells to stain the nucleus.

**Establishing the relative red/green fluorescence area**

First, cells were cropped out of the image manually to allow for analysis on each cell separately. Per repeated photoporation step, a threshold fluorescence value for both colors was determined. Pixels were labeled green if the pixel value in the green channel exceeded the green threshold. Similarly, pixels were labeled red if the red pixel value exceeded the red threshold. In this way, a pixel was labeled either black, green, red or green+red. We define the number of pixels in these categories as N_b_ N_g_ N_r_ N_gr_, respectively. For each cell, the relative green and red areas, A_g_ and A_r_ respectively, were determined by following formulas:

A_g_=(N_g_+N_gr_)/(N_g_+N_r_+N_gr_)

A_r_=(N_r_+N_gr_)/(N_g_+N_r_+N_gr_)


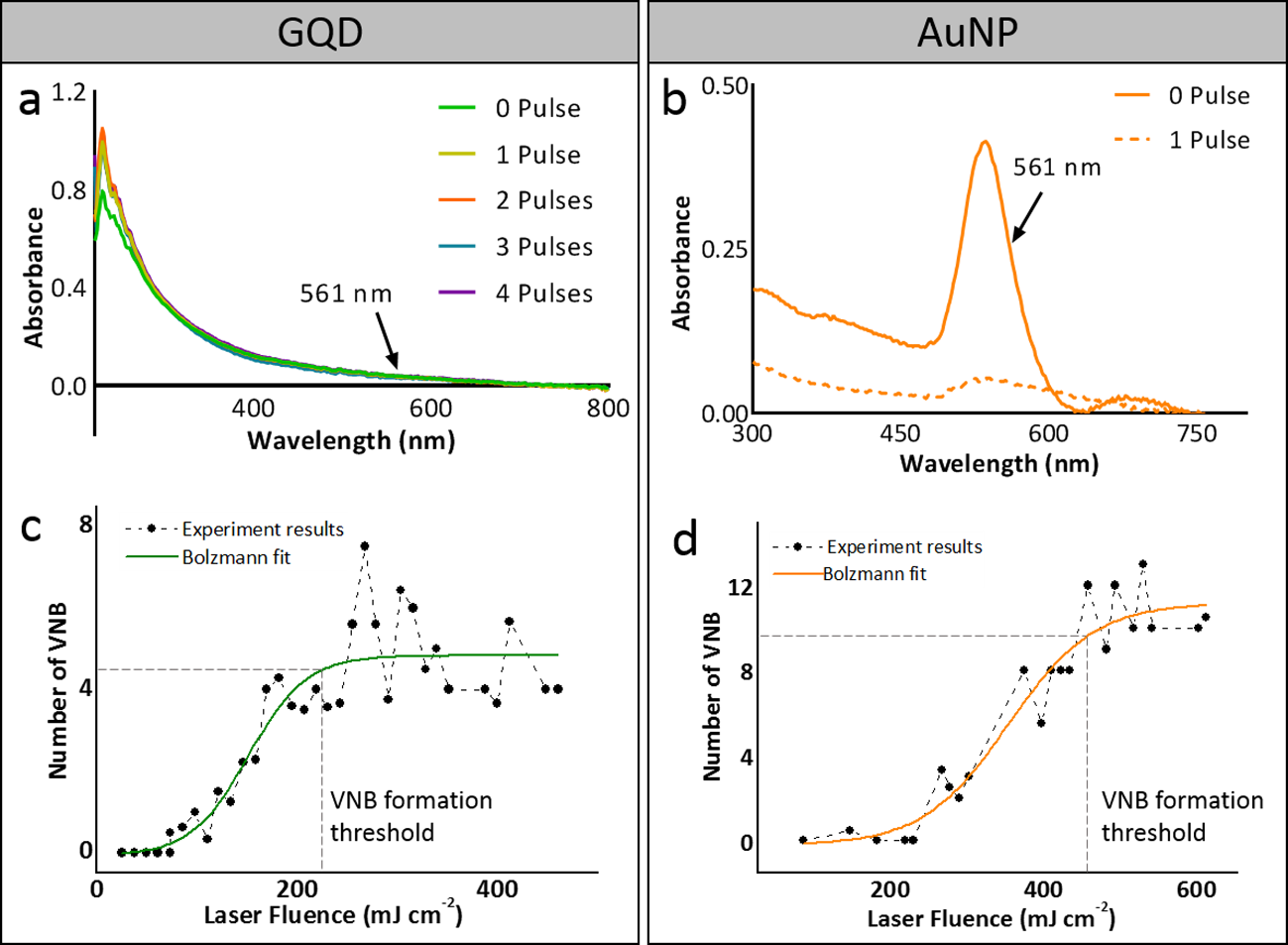


**Figure S1.** UV-Vis extinction spectra and VNB formation threshold. The UV-Vis extinction spectra of (a) 20 µg/mL GQDs and (b) 50 µg/mL AuNPs before and after being irradiated with the indicated number of laser pulses. The laser wavelength of 561 nm is indicated with a black arrow in the graphs. The extinction spectrum of GQDS remains virtually unaltered after several laser pulses while that of AuNPs dramatically changes due to particle fragmentation already after the first pulse. (c) Determination of the VNB laser fluence threshold of (c) AuNPs and (d) GQDs. The VNB threshold was defined as the laser fluence at which VNBs are formed with 90% certainty, as indicated by the dashed lines.


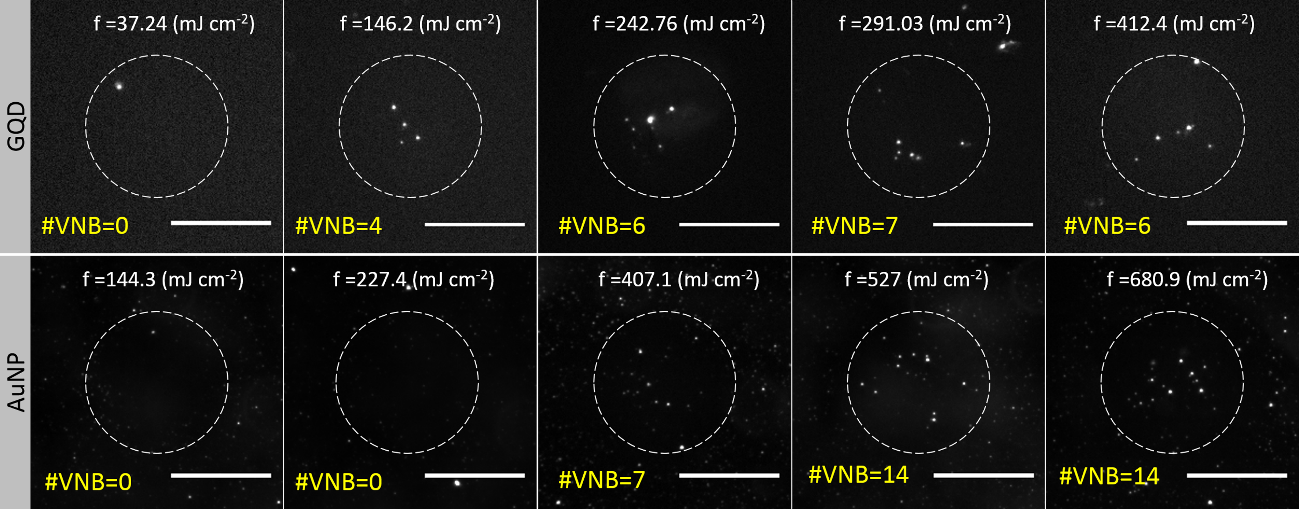


**Figure S2.** Example of dark field images to determine the VNB formation threshold. 5 µg/ml GQDs (upper row) and 0.2 µg/mL AuNPs (lower row) were diluted in Opti-MEM and irradiated by 7-ns laser pulse from low to high laser fluence. The irradiation area is indicated in the dashed circle. Scale bar is 100 µm.


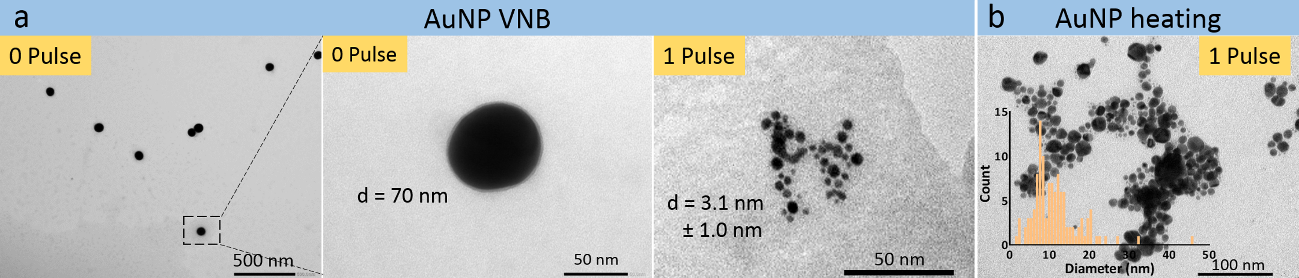


**Figure S3.** TEM images of AuNPs before laser and after laser treatment. (a) TEM images of 70 nm AuNPs before laser irradiation (0 pulse) and after irradiation with 1 laser pulse (right). The middle image shows a magnified view of a single AuNPs. After one laser pulse, the AuNPs fragment into tiny pieces of ~3.1 nm in diameter. (b) TEM images of 70 nm AuNPs after irradiation with 1 laser pulse below the VNB threshold. Also in this case AuNPs fragment into smaller pieces, although on average not as small as for irradiation above the VNB threshold.


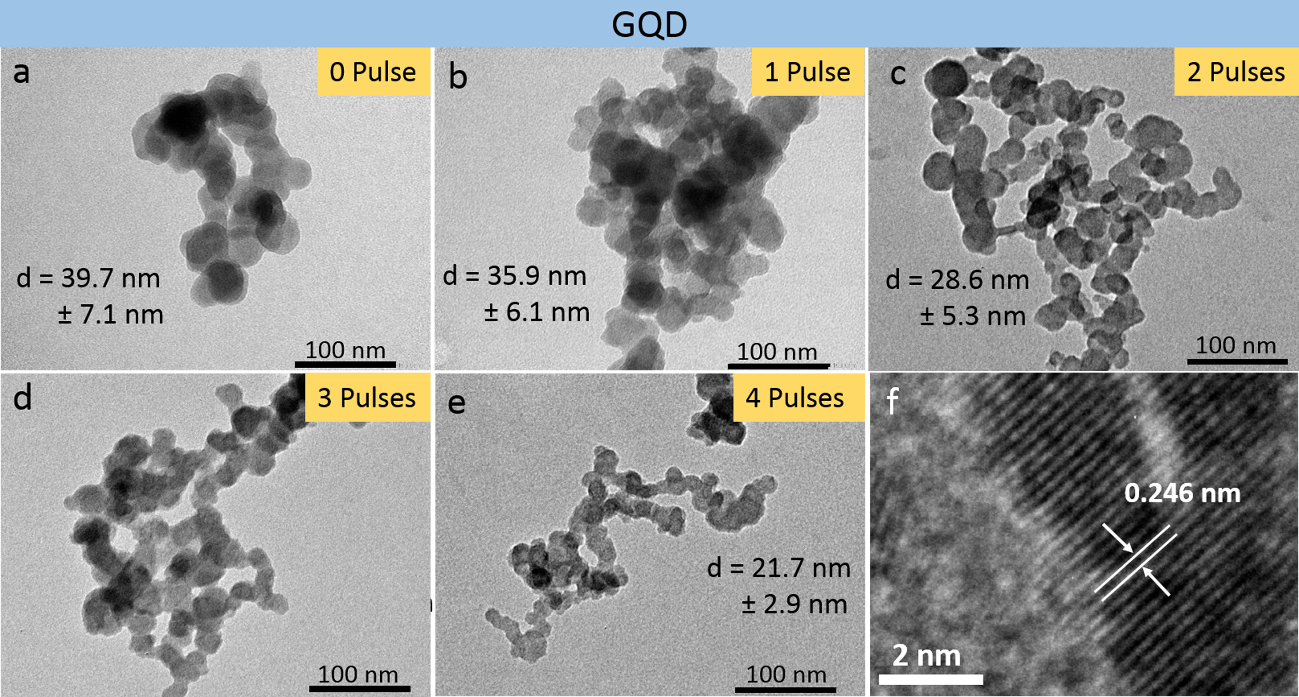


**Figure S4.** TEM images of GQDs before laser and after laser treatment. (a)TEM images of GQDs before laser irradiation (0 pulse) and (b-e) after irradiation with 1 to 4 laser pulses. The average particle diameter is indicated in the images, showing a gradual reduction with repeated irradiation. (f) HRTEM of GQDs showing a lattice spacing of 0.246 nm.


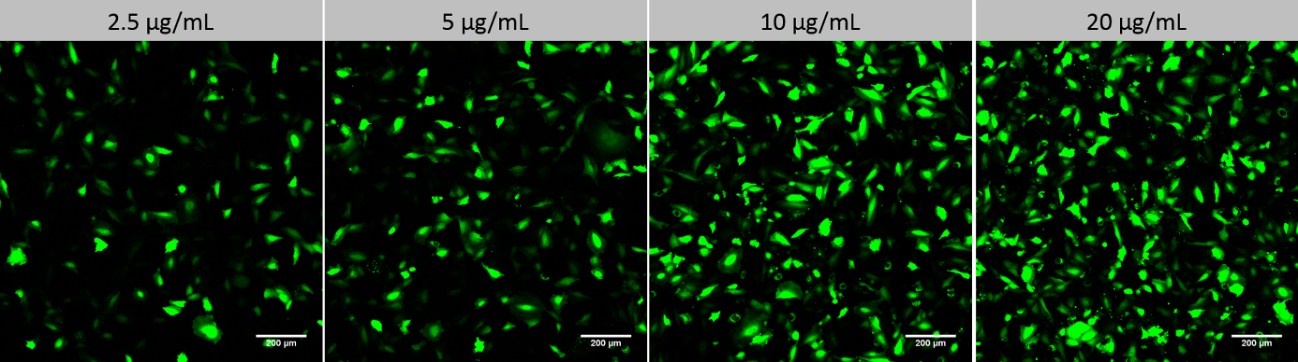


**Figure S5.** Confocal images of HeLa cells after photoporation with FD10. Cells are photoporated with a laser fluence of twice the VNB threshold with different concentrations of GQDS (2.5, 5, 10 and 20 µg/mL).


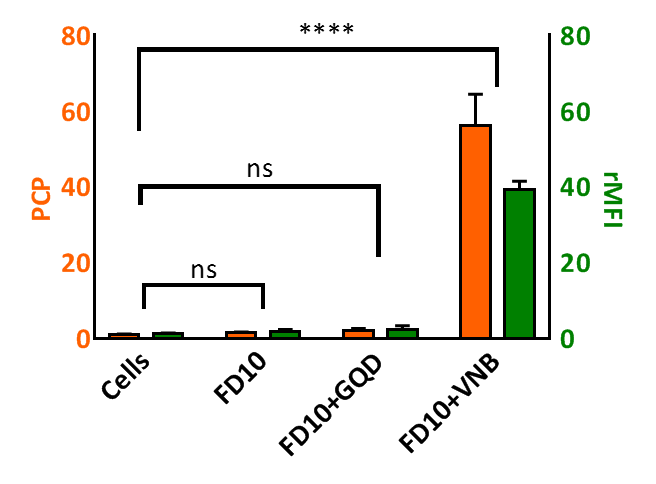


**Figure S6**. Flow cytometry results of HeLa cells labeled with FD10 in different control. It shows that FD10 is only delivered into HeLa cells after photoporation, and not by mere incubation with GQDs (FD10+GQDs) nor by itself (FD10). ****P<0.0001, n=3.


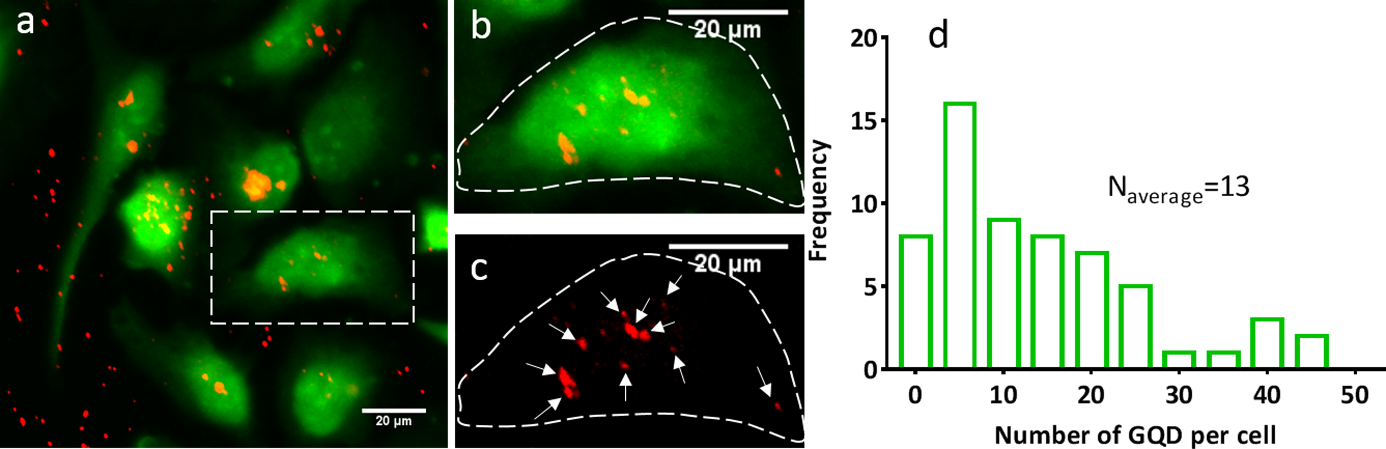


**Figure S7.** Cell-associated GQDs are visualized by fs pulsed laser illumination which causes a type of white light luminescence in carbonaceous nanoparticles**.** (a) Maximum intensity z-projected image of HeLa cells after incubation with 10 µg/ml GQDs. CellTracker staining (green) was applied to visualize cells and GQDs are false-colored in red. (b, c) Magnified view of the cell indicated by the rectangle in (a). The dashed line demarcates the cell’s boundary. (d) 60 cells were analyzed to quantify the number of GQDs per cell.

**
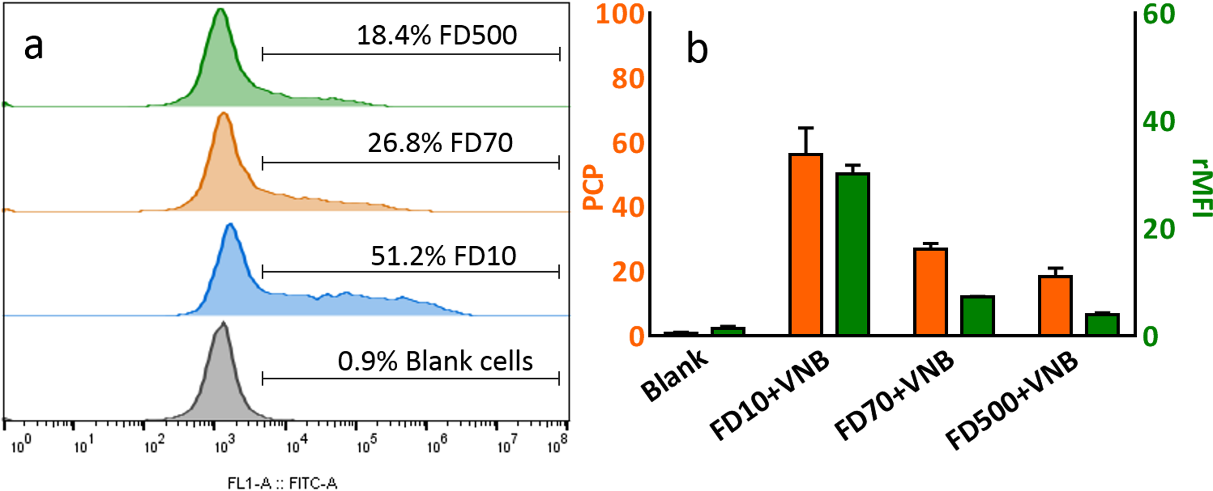
**

**Figure S8.** (a) Fluorescence distribution of HeLa cells after photoporation with FD10, FD70 and FD500. Positive cells are gated in each histogram. (b) Quantification of flow cytometry results showing the PCP (orange column) and rMFI (green column) after photoporation with FD10, FD70 and FD500. n = 3.


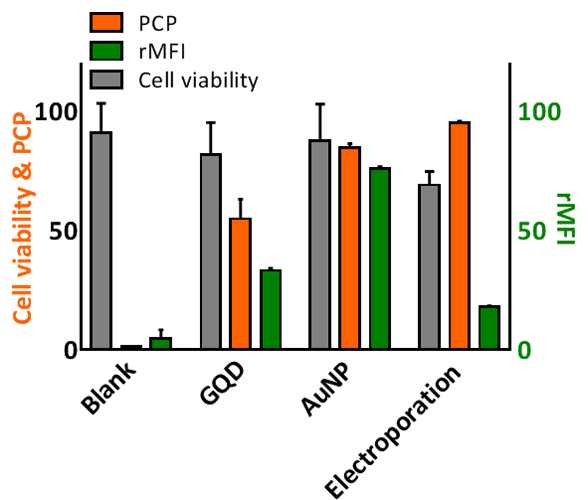


**Figure S9.** Delivery efficiency and cell viability were compared between photoporation with GQDs, photoporation with AuNPs and electroporation. n = 3.


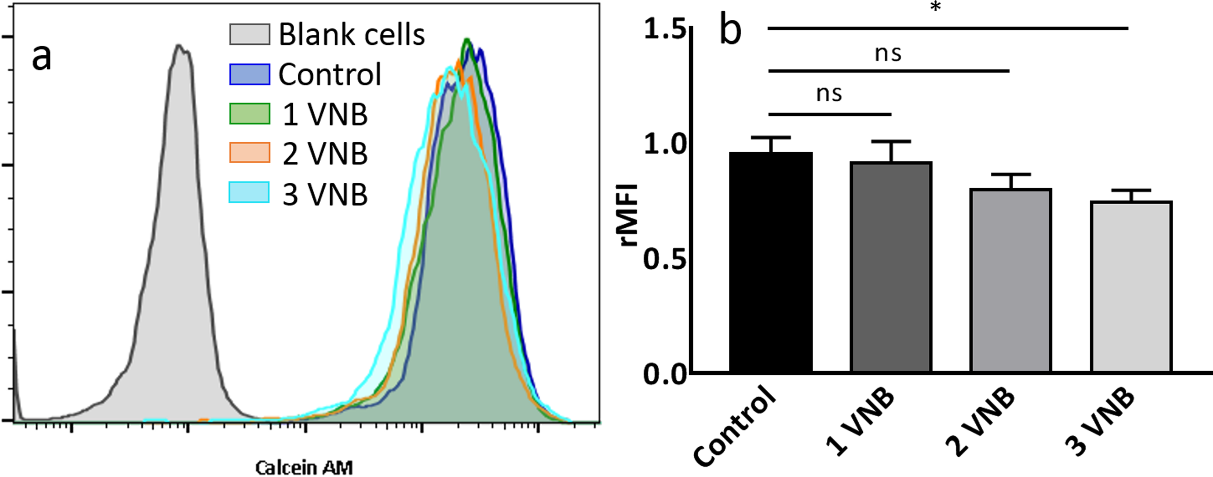


**Figure S10.** (a) Calcein AM labeled HeLa cells received photoporation treatment from 0 to 3 times. The histogram shows a slightly left-shift from control cells to 3× photoporated cells. (b) Quantification of the flow cytometry showing the rMFI after each round of photoporation. *P<0.1, n = 3.


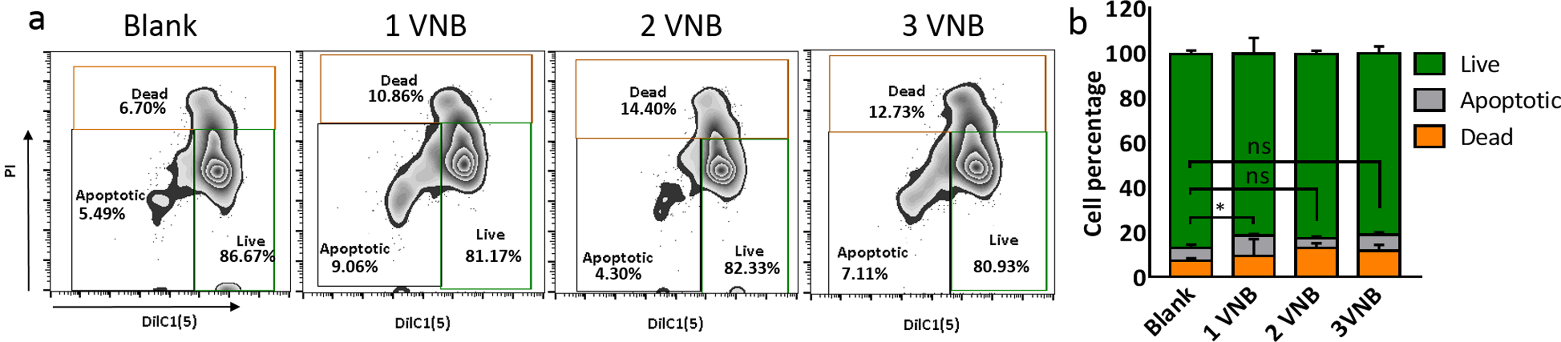


**Figure S11.** Flow cytometry results to investigate cell apoptosis after repeated photoporation with GQDs. (a) Live cells are stained with DilC1(5) (fluorescence intensity in x-axis) and dead cells are stained with PI (y-axis). The live cells are gated as indicated in the green box and dead cells in the red box. (b) Quantification of the flow cytometry shows the ratio of live cells, dead cells and apoptotic cells after photoporation. From left to right the results are untreated control cells (‘blank’), and cells treated with 1-3× photoporation. It was indicated the percentages of apoptotic cells after photoporation were not significantly different from the control group.


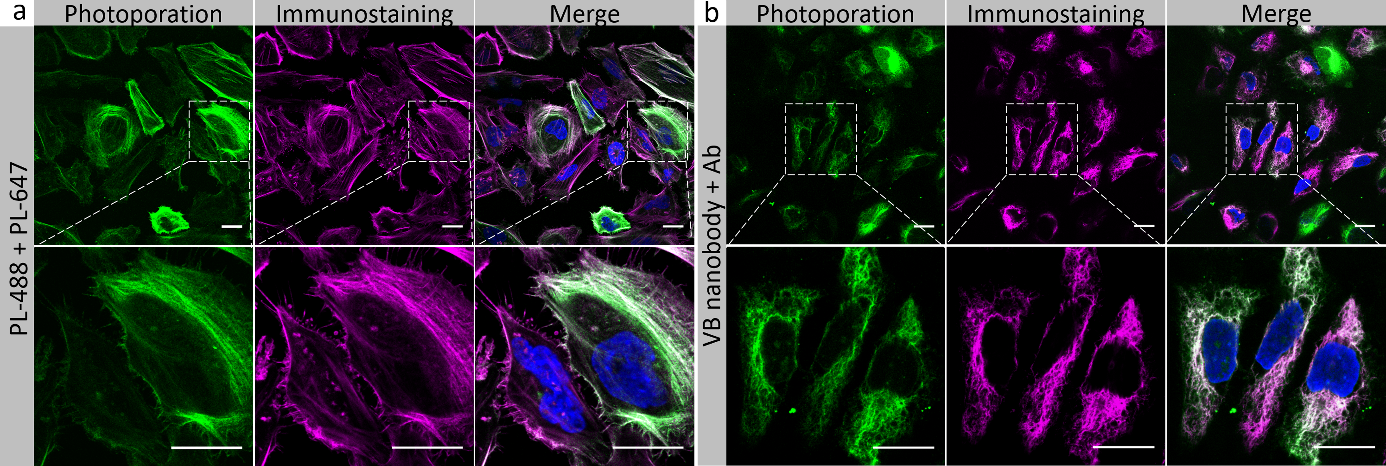


**Figure S12.** Fixed cells were co-stained with another color of fluorophore after photoporation to show specifically labeling. (a) Live HeLa cells labeled with PL-488 (green) was verified by co-staining with PL-647 (red) after cell fixation and permeabilization. (b) Live HeLa cells labeled with VB nanobody (green) was verified by co-staining with anti-vimentin primary Ab and goat anti-mouse secondary Ab Alexa Fluor 568 (red) after fixation and permeabilization. Scale bars are 20 µm.


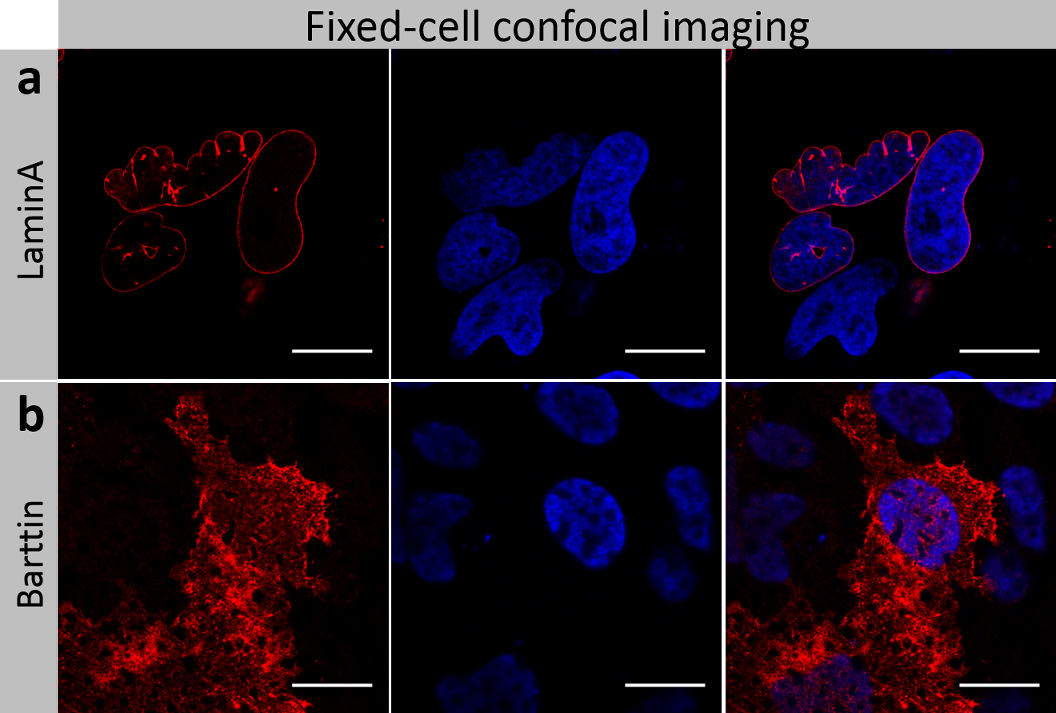


**Figure S13.** Confocal imaging of fixed HeLa cells labeled with SNAP-Surface® Alexa Fluor® 647 (red fluorescence) and counterstained with the nuclear stain Hoechst (blue). (a) SNAP^LaminA^ and (b) SNAP^Barttin^ transfected cells were fixed and permeablized and subsequently labeled with the SNAP ligand. Scale bars are 20 µm.


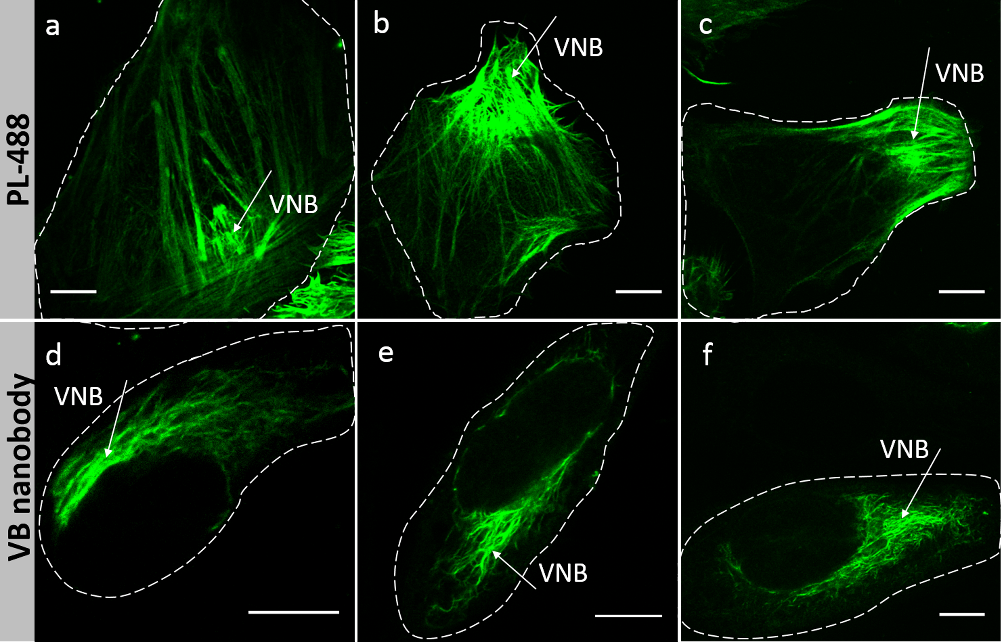


**Figure S14.** Examples of heterogeneous intracellular labeling after 1× photoporation. (a-c) F-actin in living HeLa cells were labeled with PL-488. (d-f) Vimentin in living HeLa cells were labeled with VL nanobody. The staining is most pronounced close to the area where pores were formed by VNB photoporation. Scale bars are 10 µm.

**Supplementary Movies**

**Suppl. Movie 1. Dark-field imaging of VNBs from discrete AuNPs in Opti-MEM.** 7 ns laser pulses are applied 4 times in the indicated circular area. Each time a dark-field image is recorded before and during laser irradiation. Due to particle fragmentation, VNBs can be formed only once in case of AuNPs. Note that individual VNB are only visible during 1 frame due to their very short life time which is in the order of 10-100 ns.

**Suppl. Movie 2. Dark-field imaging of VNBs from discrete GQDs in Opti-MEM.** The same experiment is performed as for Movie 1. As GQDs are more resistant to laser irradiation compared to AuNPs, VNBs can still be seen after each of the four applied laser pulses.

**Suppl. Movie 3. Dark-field confocal microscopy imaging of VNBs from AuNPs imbedded in a living HeLa cell membrane.** Cells were incubated with AuNPs diluted in Opti-MEM at 37°C for 30 min. Laser pulses were generated 4 times by a pulse generator as shown in the white circle area. Images were captured before and during the pulse generation.

**Suppl. Movie 4. Dark-field confocal microscopy imaging of VNBs from GQDs imbedded in a living HeLa cell membrane.** Cells were incubated with AuNPs diluted in Opti-MEM at 37°C for 30 min. laser pulses were generated 4 times in the white circle area. Images were captured before and during the pulse generation.

**Suppl. Movie 5. Time-lapse confocal microscopy imaging of living cells labeled of actin filament with PL-488 (green) delivered by photoporation.** The nuclei (blue) are counterstained with Hoechst. The interval time was 3 min and 19 frames in total were taken.

**Suppl. Movie 6. Z-stack imaging of a living HeLa cell labeled with VB nanobody after 1× photoporation.** 40 µg/mL VB nanobody were delivered into cells by 1× photoporation. A z-stack image of live HeLa cell was taken with a z-step of 0.125 µm and total 6.75 µm.

**Suppl. Movie 7. Time-lapse confocal imaging of live cells labeled with VB nanobody delivered by photoporation.** The nuclei (blue) were labeled by Hoechst. The interval time was 3 min and 20 frames in total were taken.

**Suppl. Movie 8. Time-lapse Airyscan superresolution microscopy imaging of living cells.** HeLa cells transfected with LaminA SNAP-tag pDNA, and photoporated in the presence of SNAP-Surface® Alexa Fluor® 647. The nuclei (blue) are stained with Hoechst. 30 frames were taken with a time interval of 1 min.

**Suppl. Movie 9. Time-lapse TIRF microscopy imaging of living cells.** HeLa cells were transfected with Barttin SNAP-tag pDNA and photoporated in presence of SNAP-Surface® Alexa Fluor® 647. 90 frames were taken with an interval time of 1 sec.

**References**

1 Lukianova-Hleb, E., Hu, Y., Latterini, L., Tarpani, L., Lee, S. et al. Plasmonic nanobubbles as transient vapor nanobubbles generated around plasmonic nanoparticles. *ACS nano* 2010; **4:** 2109.

2 Lapotko, D. & Lukianova, E. Laser-induced micro-bubbles in cells. *International Journal of Heat and Mass Transfer* 2005; **48:** 227-234.

3 Lapotko, D. Optical excitation and detection of vapor bubbles around plasmonic nanoparticles. *Optics Express* 2009; **17:** 2538-2556.

4 Singh, S. K., Dhavale, V. M., Boukherroub, R., Kurungot, S. & Szunerits, S. N-doped porous reduced graphene oxide as an efficient electrode material for high performance flexible solid-state supercapacitor. *Applied Materials* *Today* 2017; **8:** 141-149.

5 Bové, H., Steuwe, C., Fron, E., Slenders, E., D’Haen, J. et al. Biocompatible Label-Free Detection of Carbon Black Particles by Femtosecond Pulsed Laser Microscopy. *Nano letters* 2016; **16:** 3173-3178.
